# High expression of *FAM13A* was associated with increasing the liver cirrhosis risk

**DOI:** 10.1002/mgg3.543

**Published:** 2019-01-02

**Authors:** Yingai Zhang, Shunlan Wang, Chan Wang, Jingchuan Xiao, Shufang Zhang, Hailong Zhou

**Affiliations:** ^1^ Institute of Tropical Agriculture and Forestry Hainan University Haikou China; ^2^ Central Laboratory Central South University Xiangya School of Medicine Affiliated Haikou Hospital Haikou China

**Keywords:** *FAM13A*, genetics polymorphism, immunohistochemistry, liver cirrhosis, SNP

## Abstract

**Aim:**

Liver cirrhosis is a consequence of chronic liver disease, and it may be caused by multiple influences of both genetic and environmental factors. Family with sequence similarity 13 member A (*FAM13A*) has been previously associated with lung function in several lung diseases, including chronic obstructive pulmonary disease, asthma, lung cancer, and pulmonary fibrosis. The aim of this study was to explore whether *FAM13A *polymorphisms confer susceptibility to liver cirrhosis.

**Methods:**

*FAM13A* expression was evaluated in liver cirrhosis tissues by immunohistochemistry staining. The relationship between *FAM13A* gene polymorphism and liver cirrhosis was determined by association analysis. The genotypes were assessed in the Agena MassARRAY platform. Statistical analysis was performed using chi‐squared test/Fisher's exact test, genetic model analysis, and haplotype analysis.

**Results:**

The results showed that the expression of *FAM13A* is obvious higher in the liver cirrhosis tissue cells than in the normal liver tissue cells. Moreover, association analysis results indicated that the minor allele “A” of rs3017895 was positively associated with high risk of liver cirrhosis in the allele model by the chi‐squared test (OR = 1.32, 95%CI = 1.03–1.68, *p = *0.028). Logistic regression analyses revealed that the risk of liver cirrhosis was significantly higher in subjects with the G/A‐G/G genotype of rs3017895 than those with A/A genotype under the dominant model and log additive model, and the T/A‐A/A genotype of rs1059122 was positively associated with higher liver cirrhosis than T/T genotype based on dominant model respectively. In addition, haplotype analysis showed that the G‐A haplotype of rs3017895‐rs1059122 of the *FAM13A* gene significantly increased the risk of liver cirrhosis.

**Conclusion:**

Our findings demonstrated that the high expression of *FAM13A* may be associated with an increased risk of liver cirrhosis.

## 1 INTRODUCTION

Liver cirrhosis caused by long‐term or repeated damage to liver parenchyma by various factors represents the main complication of chronic liver disease, which leads progressive liver failure, eventually to hepatocarcinoma (Chang et al., 2015; Schuppan & Afdhal, 2008). Liver cirrhosis is a severe public health problem worldwide, which is correlated with higher morbidity and mortality worldwide (Chung, Jo, Chung, & Kim, 2018). The pathogenesis of liver cirrhosis remains unclear; although the common risk factors, such as diet and physical inactivity for NAFLD, excessive alcohol intake for alcoholic liver disease (ALD), or infection for chronic viral hepatitis, can be identified in the majority of patients, there is a small percentage of patients with no identifiable risk factors (Xiong, Liu, & Zhang, 2018; Zocco et al., 2009).

Several studies have shown that the etiology and pathogenesis of Cirrhosis were likely to comprise a multifactorial disorder resulting from environmental and genetic factors and their interaction (Ramos‐Lopez, Martinez‐Lopez, Roman, Fierro, & Panduro, 2015). Genetic factors play a key role in determining the inter‐individual susceptibility toward liver diseases, including the cirrhosis (He, Deng, & Luo, 2015; Sheneef et al., 2017). For example, several study showed that Cytochrome certain gene polymorphisms was a molecular genetic marker of liver cirrhosis and hepatocellular carcinoma progression, such as *CYP2E1*, *ADH2*, *TGF‐β,* and interleukin family related genes (*IL‐10*, *IL‐6*, *IL‐28B,* and *IL‐1*; Cichoz‐Lach, Partycka, Nesina, Celiński, & Słomka, 2006; Guo, Jin, & Sun, 2015; Wu, Zeng, Gong, Chen, & Chen, 2013).

The biological function of the *FAM13A* (Family with sequence similarity 13 member A, OMIMl: 613299) gene is poorly understood. Researches revealed that the *FAM13A *gene has a putative role in signal transduction, which points out the potential importance of the *FAM13A* in diseases (Cohen et al., 2004). Recent genome‐wide association studies reveal that the *FAM13A* gene was associated with a variety of lung diseases, including chronic obstructive pulmonary disease, asthma, lung cancer, and pulmonary fibrosis (Corvol, Hodges, Drumm, & Guillot, 2014; Hawkins & Mora, 2017). However, no studies have investigated the association between genetic variants in* FAM13A* and the risk of liver cirrhosis. Therefore, we performed a case–control study to analyze the association between the *FAM13A *and the risk of liver cirrhosis.

## 2 MATERIALS AND METHODS

### 2.1 Subject recruitment and ethics committee statement

Two hundred and sixty primary liver cirrhosis patients and 384 controls were enrolled in this study, all of whom were genetically unrelated Han Chinese. All participants were recruited from Haikou People's Hospital. All primary liver cirrhosis patients were diagnosed histopathologically or based on the specific morphological criteria of liver cirrhosis with ultrasound, computed tomography, or magnetic resonance imaging. Control group were age‐ and gender‐matched healthy subjects without liver cirrhosis or other diseases. All subjects were examined without history of cancer or other diseases.

Informed consents were obtained from participants, and all of them were agree to this study. The use of human tissue and the protocol in this study were strictly conformed to the principles expressed in the Declaration of Helsinki, and this study was carried out with approval from the ethics committee of the Haikou People's Hospital.

### 2.2 Immunohistochemistry

Specimens obtained from surgical resection were fixed in 10% formalin prior to being processed in paraffin. Hematoxylin–eosin‐stained sections were reviewed by a pathologist, and a representative section was selected for immunohistochemical analysis. The sections were stained within 5 days of cutting using an Autostainer Link48 (Dako, USA) in strict accordance with the manufacturer's instructions. Immunohistochemical staining was performed using an EnVisionTM HRP‐polymer anti‐mouse IHC Kit (K8002; Dake BioTECH, USA, Inc) according to the manufacturer's guidelines. The primary antibodies specific to *FAM13A* (anti‐*FAM13A*, 1:500; HPA038109) were obtained from Sigma‐Aldrich (St. Louis, MO, USA). Finally, we observed the images of the scanned tissue slices through Aperio ImageScope (Version 11.1.2.752).

### 2.3 SNP selection and genotyping

Peripheral blood samples were collected in an anti‐coagulation tube and stored at −80°C until detection before subjects had received other therapies. Based on the manufacturer's instructions of the GoldMag‐Mini Purification Kit (GoldMag Co.Ltd. Xi'an city, China), genomic DNA was isolated from blood leukocytes samples. DNA concentrations were measured using the NanoDrop 2000 (Thermo Fisher Scientific, Waltham, MA, USA).

A total of two SNPs at the 3′‐UTR of *FAM13A *were selected at a minor allele frequency >5% in the 1,000 Genomes Project (https://www.internationalgenome.org/). The primers were designed online (https://agenacx.com/online-tools/). The Agena Bioscience platform (https://www.agenabio.com) based on the matrix‐assisted laser desorption/ionization time‐of‐flight (MALDI‐TOF) primer extension assay was used to genotype two SNPs (rs3017895 and rs1059122).

### 2.4 Statistical analysis

SPSS 19.0 (SPSS, Chicago, IL, USA) was used to perform statistical analyses. Genotyping results were output by Agena Bioscience TYPER version software 4.0. The Pearson's chi‐squared test and independent‐samples Student's *t* test were applied to assess the differences in the distribution of demographic characteristics between cases and controls. Fisher's exact tests for Hardy–Weinberg equilibrium (HWE) were performed by comparing the observed and expected genotype frequencies to calculate the genotype frequencies among the controls. Odds ratios (OR) and 95% confidence intervals (CI) were calculated to estimate the association between *FAM13A* gene and the risk of Cirrhosis using unconditional logistic regression analysis with or without adjustment for age and gender. The SNP effects were fitted under four models of inheritance: codominant, dominant, recessive, and a log‐additive model by PLINK software (Version 1.07). Haplotype construction and genetic association of polymorphism loci were assessed using the Haploview software package (version 4.2) and the SHEsis software (https://analysis.bio-x.cn/myAnalysis.php). All *p *values of statistical tests were two‐sided, and *p < *0.05 was considered as statistically significant.

## 3 RESULT

### 3.1 High expression of *FAM13A* in the primary liver cirrhosis

**Figure 1:**
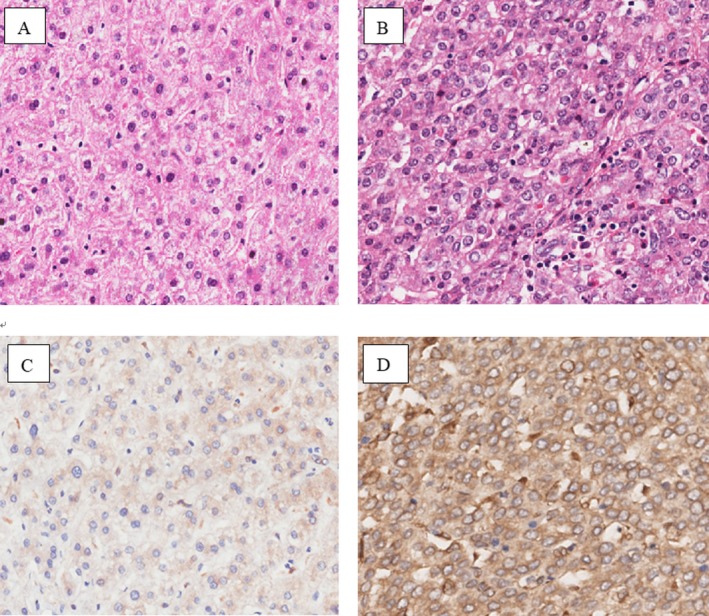
Morphological observation of normal liver tissue (a) and cirrhotic tissue (b), and the expression of *FAM13A *in normal liver tissue (c) and cirrhotic liver tissue (d)

As shown in Figure 1, we observed the morphological observation of normal liver tissue cells and liver cirrhosis tissue cells by hematoxylin–eosin (HE) staining showed that there are obvious differences in morphology between liver cirrhosis tissue cells and normal liver tissue cells under the electron microscope (10×), and the size and shape of liver cirrhosis tissue cells are inconsistent, and the volume of nucleus increased (Figure 1a–b). Representative photomicrographs of staining intensity of *FAM13A* expression in liver cirrhosis tissue cells are shown in Figure 1c–d. Compared with Figure 1c, *FAM13A* expression is obvious enhanced in liver cirrhosis tissue cells (Figure 1d).

### 3.2 Characteristics of study participants

Basic clinical characteristics of 260 cirrhosis patients and 384 disease‐free controls in this study were provided in Table 1. In patients’ characteristics, age showed no significant difference between case (51.14 ± 11.55) and controls (51.16 ± 11.49) (*p = *0.722). And, there was no significant difference in gender distribution (case: male 172, female 88; control: male 278, female 106; *p = *0.123).

**Table 1:** The characteristic of case and control

Variable	Case	Control	*p*
Age (year, *SD*)	51.14 ± 11.55	51.16 ± 11.49	0.722^a^
Gender			
Male	172 (66.15%)	278 (72.4%)	0.123^b^
Female	88 (33.85%)	106 (27.6%)	

a *p* values were calculated by Student's *t* tests;

b *p* values were calculated from two‐sided chi‐squared tests.

### 3.3 Association between *FAM13A* SNPs and the risk of cirrhosis

Rs3017895 and rs1059122 in the 3′‐UTR of *FAM13A* were selected. Position, alleles and minor allele frequency of these two SNPs were showed in Table 2. In control groups, two SNPs were in line with HWE (*p = *0.435 for rs3017895, *p = *0.179 for rs1059122). Pearson's chi‐squared test was used to assess the association between SNP variants and the risk of liver cirrhosis. The minor allele “A” of rs3017895 was associated with significant higher the risk of liver cirrhosis (OR = 1.315, 95%CI = 1.03–1.68, *p = *0.028).

**Table 2:** Basic information of candidate SNPs and minor allele frequency between cases and controls

SNP rs#	Chromosome	Position	Role	Alleles A/B	Gene(s)	MAF		*p*_‐HWE_	OR	95%CI	*p* a
						Case	Control				
rs3017895	4	89649491	3′‐UTR	A/T	*FAM13A*	0.322	0.266	0.435	**1.32**	**1.03–1.68**	**0.028** *
rs1059122	4	89647424	3′‐UTR	G/A	*FAM13A*	0.523	0.473	0.179	1.22	0.97–1.53	0.081

Note

Alleles A/B: Minor/major alleles; CI: confidence interval; HWE: Hardy–Weinberg equilibrium; MAF, minor allele frequency; OR: odds ratio; SNP: single‐nucleotide polymorphism.

Bold highlights the value of *p* and OR (95%CI) with statistical significance.

a *p *values were calculated using two‐sided chi‐squared test (the major allele of each SNP was a reference allele).

* *p ≤ *0.05 indicates statistical significance.

After adjustment for age and gender, four genotypes model of *FAM13A* polymorphisms were shown in Table 3. There was a significant differences between the groups. Dominant model analyses revealed that the risk of liver cirrhosis was significantly higher in subjects with the G/A‐G/G genotype of rs3017895 than those with A/A genotype (OR = 1.42, 95%CI = 1.03–1.95, *p* = 0.03), and there was also a significant difference under the log‐additive model (OR =1.31, 95%CI = 1.03–1.67, *p* = 0.03). And the T/A‐A/A genotype of rs1059122 was positively associated with higher liver cirrhosis under dominant model than T/T genotype (OR = 1.48, 95%CI = 1.03–2.15, *p* = 0.034).

**Table 3:** Association between candidate SNPs and the risk of cirrhosis under genotype models

SNP	Model	Genotype	Genotype frequency		OR (95% CI)	*p*‐Value	AIC	BIC
			Case	Control				
rs3017895	Codominant	A/A	119 (46%)	210 (54.7%)	1	0.084	868	881.4
		G/A	113 (43.6%)	144 (37.5%)	1.38 (0.99–1.93)			
		G/G	27 (10.4%)	30 (7.8%)	1.59 (0.90–2.80)			
	Dominant	A/A	119 (46%)	210 (54.7%)	1	**0.03** *	866.2	875.1
		G/A‐G/G	140 (54%)	174 (45.3%)	**1.42 (1.03–1.95)**			
	Recessive	A/A‐G/A	232 (89.6%)	354 (92.2%)	1	0.26	869.6	878.6
		G/G	27 (10.4%)	30 (7.8%)	1.37 (0.80–2.37)			
	Log‐additive	—	—	—	**1.31 (1.03–1.67)**	**0.03** *	866.2	875.2
rs1059122	Codominant	T/T	57 (22%)	111 (29.5%)	1	0.1	860.1	873.5
		T/A	133 (51.4%)	174 (46.3%)	1.49 (1.01–2.20)			
		A/A	69 (26.6%)	91 (24.2%)	1.48 (0.94–2.31)			
	Dominant	T/T	57 (22%)	111 (29.5%)	1	**0.034** *	858.1	867
		T/A‐A/A	202 (78%)	265 (70.5%)	**1.48 (1.03–2.15)**			
	Recessive	T/T‐T/A	190 (73.4%)	285 (75.8%)	1	0.49	862.1	871
		A/A	69 (26.6%)	91 (24.2%)	1.14 (0.79–1.63)			
	Log‐additive	—	—	—	1.21 (0.97–1.51)	0.086	859.7	868.6

Note

AIC: Akaike's Information criterion; BIC: Bayesian Information criterion; CI: confidence interval; OR: odds ratios.

Bold highlights the value of *p* and OR (95%CI) with statistical significance.

*p *values were calculated from Wald's test adjusted for age and sex.

* *p ≤ *0.05 indicates statistical significance.

### 3.4 Haplotype association

**Figure 2:**
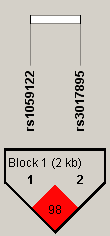
Linkage disequilibrium (LD) plots containing five SNPs from *FAM13A*

Finally, allele frequency data from all subjects were used to do the linkage disequilibrium (LD) block, and we found a strong LD between rs3017895 and rs1059122 with D′ = 0.98 (Figure 2). The results of the association between haplotypes and the liver cirrhosis risk were shown in Table 4. There were three haplotypes “A‐T,” “G‐A,” and “A‐A”; however, only the “G‐A” haplotype was significantly associated with the liver cirrhosis risk by Pearson's chi‐squared test (*p = *0.021). After unconditional logistic regression analysis adjustment for age and gender, the “G‐A” haplotype was remained significantly increasing the liver cirrhosis risk (OR = 1.34, 95%CI = 1.04–1.73, *p = *0.026).

**Table 4:** The haplotype of two SNPs (rs3017895 and rs1059122) in *FAM13A* and the liver cirrhosis risk

	rs3017895	rs1059122	Haplotype frequency		*p* a	OR (95%CI)	*p* b
			Case	Control			
1	A	T	0.476	0.522	0.106	1	‐
2	G	A	0.322	0.263	0.021*	**1.34 (1.04–1.73)**	**0.026** *
3	A	A	0.202	0.212	0.643	1.03 (0.77–1.38)	0.83

Bold highlights the value of *p* and OR (95%CI) with statistical significance.

a *p* values were calculated by two side chi‐squared test;

b *p* values were calculated by Wald test adjusted by gender and age;

* *p* < 0.05 indicates statistical significance.

## 4 DISCUSSION

Liver cirrhosis occurs as a consequence of many chronic liver diseases that are prevalent worldwide. Several studies showed that the etiology and pathogenesis of Cirrhosis were likely to comprise a multifactorial disorder resulting from environmental and genetic factors and their interaction. In the present case–control study, we first used immunohistochemistry (IHC) to detect the expression of the *FAM13A* gene in normal liver tissue and cirrhotic tissue. We found that the expression level of *FAM13A* in liver cirrhosis tissues was significantly higher than the normal tissues. We predicted that this gene may be a risky gene for liver cirrhosis. Subsequently, two SNPs in this gene are screened for association analysis. The results demonstrate that *FAM13A *genetic polymorphisms are associated with cirrhosis risk, which indicate that the *FAM13A* gene may play an important role in the risk of liver cirrhosis in the Chinese population.

The *FAM13A* gene, mapped to chromosome 4q22, contains 25 exons spanning about 332 kb, is an important switch component of the cellular pathways controlling cell cycle and proliferation (Cohen et al., 2004; Jin et al., 2015). *FAM13A* is a modifier gene of cystic fibrosis lung phenotype regulating rhoa activity, actin cytoskeleton dynamics, and epithelial–mesenchymal transition (Corvol et al., 2018). The *FAM13A *gene was participated in the Wnt pathway, which is an important part in regulating the adult tissue homeostasis (Zhang et al., 2008). However, a dysregulation of the Wnt pathway could cause grave consequences like tumourigenesis and other severe diseases, which points out the potential importance of the *FAM13A* in diseases (Kikuchi, 2000; Krishnamurthy & Kurzrock, 2018). One study reported the *FAM13A* interacts with PP2A and β‐catenin to regulate β‐catenin protein stability it could promote cell proliferation, at the same time, the result also showed *FAM13A* determines susceptibility to emphysema by regulating β‐catenin signaling (Jiang et al., 2016). Recent genome‐wide association studies revealed that the *FAM13A* gene was associated with human lung function and a variety of lung diseases, including chronic obstructive pulmonary disease, asthma, lung cancer, and pulmonary fibrosis (Eisenhut et al., 2016; Hirano et al., 2017; Wang et al., 2013). In the meanwhile, Eisenhut et al. (2017) denoted *FAM13A* was associated with non‐small cell lung cancer (NSCLC) progression and controls tumor cell proliferation and survival. Hirano et al. (2017) reported the polymorphism of *FAM13A* gene had a significant association with the susceptibility to idiopathic pulmonary fibrosis, with severity of lung function impairment and with poor prognosis. However, studies about the *FAM13A* and other diseases have not been reported, including liver cirrhosis. Therefore, it was necessary to find genetic markers to predict people who were susceptible to liver diseases.

In our study, we investigated two SNPs in *FAM13A*, including rs3017895 and rs1059122, and we find the SNPs in *FAM13A *gene were associated with an increased the risk liver cirrhosis. As far as we know, we are the first to report the association between *FAM13A* polymorphisms and liver cirrhosis risk, but the results identified here should be confirmed in further studies. However, some potential limitations of our current study should be considered when decipher the results. A limited number of studies has been conducted on the FAM13A gene expression to identify a potential novel biomarker for liver cirrhosis. Further functional studies and larger population‐based prospective studies are required in order to understand the genetic factors underlying liver cirrhosis in the subsequent research.

## 5 CONCLUSION

Our results indicate that the expression level of *FAM13A* in liver cirrhosis tissues was significantly higher than the normal tissues. The polymorphisms of rs3017895 and the rs1059122 in *FAM13A* are associated with an increased risk of liver cirrhosis.

## CONFLICTS OF INTEREST

The authors have no conflicts of interest to report.
